# High Fluoride Ingestion Impairs Bone Fracture Healing by Attenuating M2 Macrophage Differentiation

**DOI:** 10.3389/fbioe.2022.791433

**Published:** 2022-05-20

**Authors:** Chengcheng Du, Pengcheng Xiao, Shengqiang Gao, Shengwen Chen, Bowen Chen, Wei Huang, Chen Zhao

**Affiliations:** Department of Orthopedics, The First Affiliated Hospital of Chongqing Medical University, Orthopedic Laboratory of Chongqing Medical University, Chongqing, China

**Keywords:** fluorosis, excessive fluoride intake, fracture, M2 macrophages, fracture healing

## Abstract

Fluorosis is still endemic in at least 25 countries around the world. In this study, we investigated the effect of high fluoride intake on fracture healing. Our *in vitro* experiments found that fluoride inhibited the osteogenic and angiogenic differentiation of MSCs in a dose-dependent manner. By constructing a bone fracture model, we found that high fluoride intake influences bone fracture by attenuating endochondral ossification and angiogenesis. In the mechanism, we clarified that high fluoride inhibits M2 differentiation rather than M1 differentiation in the fracture area, which may contribute to the delayed healing of the fracture. These findings provide an essential reference for the clinical treatment of bone fracture patients with a history of high fluoride intake or skeletal fluorosis patients.

## Introduction

Long-term and excess fluoride consumption induces disturbed homeostasis of the bone and a series of chronic systemic diseases (Erdal and Buchanan, 2005). The World Health Organization has stated that the maximum safe limit of fluoride in drinking water is 1.5 ppm; however, more than 50 countries have high fluoride levels in drinking water (Rango et al., 2012; Perumal et al., 2013; Yu et al., 2018; Zhou et al., 2019). Fluorosis is endemic in at least 25 countries around the world, of which India and China are the most affected. East Africa and North Africa, Mexico, and Latin America are also endemic areas for fluorosis (Pramanik and Saha, 2017). Men are usually more likely to have severe fluorosis than women, and studies have shown that childhood fluorosis can affect bone development (Jha et al., 2011; Saeed et al., 2020). Skeletal fluorosis (SF) is the major clinical manifestation caused by the excessive accumulation of fluoride (Srivastava and Flora, 2020). The main pathological features of SF include joint pain, muscle weakness, and skeletal deformities (Wei et al., 2019). Clinical treatment is challenging when fluorosis patients have co-morbidities with osteoarthritis, fracture, or other severe bone trauma (Everett, 2011). However, no approaches for decreasing the concentration of fluoride in the body have been developed; these therapies may have inhibitory effects but cannot cure SF (Johnston and Strobel, 2020). Therefore, it is important to further understand the effect of fluoride on bone fracture healing for clinical treatment.

Fluoride directly affects the *bones via* two main mechanisms (Ciosek et al., 2021). In mineralized tissues, fluoride is incorporated into apatite crystals in the process of ion exchange, which leads to the formation of fluorapatite (Johnston and Strobel, 2020; Ciosek et al., 2021). Such conversion results in changes in crystallinity and a reduction in mechanical properties (Ciosek et al., 2021). In bioactive tissue, fluoride also stimulates osteoblasts and osteocytes in a concentration-dependent mechanism (Jiang et al., 2020a; Chu et al., 2020). These studies suggest that fluoride is important in bone metabolism, but there is no direct evidence that has reported the effects of excess fluoride on fracture healing.

Normally, 10–15% of these patients have unsatisfactory fracture healing (Hak et al., 2014; Loi et al., 2016; Ho-Shui-Ling et al., 2018). Unfortunately, when combined with bone metabolic diseases such as SF, the rate of delayed or non-union fractures was increased (Nampei and Hashimoto, 2009; Ellegaard et al., 2010; Marin et al., 2018). The events during the fracture healing cascade include the initial inflammatory phase, hematoma formation, progenitor cell recruitment, formation of an intermediate callus, maturation of the callus, and the final remodeling of the bony callus to the original bone’s structure and shape (Alexander et al., 2011; Einhorn and Gerstenfeld, 2015). Commonly, failure in the bone healing process is due to insufficient numbers of progenitor cells and disrupted vascularization (Pajarinen et al., 2019). Macrophages play an important role in recruiting progenitor cells and angiogenesis (Schlundt et al., 2018; Pajarinen et al., 2019). Macrophages may acquire distinct phenotypes with proinflammatory (M1) or anti-inflammatory (M2) functions at the fracture site, a phenomenon known as macrophage polarization (Schlundt et al., 2018). Up to now, the effect of fluoride on the polarization of macrophages and the M1/M2 ratio has been unknown.

In the present study, we aimed to explore whether excessive fluoride ingestion has effects on bone fracture healing. We first found that excessive fluoride exposure delayed bone fracture healing, especially at the stage of maturation of the callus. Subsequently, we presented *in vitro* evidence to support that high fluoride concentration can effectively inhibit M2 differentiation. The findings in this study suggest that the decrease of M2 may be related to delayed fracture healing and provide a new research direction for the treatment of skeletal fluorosis.

## Materials and Methods

### Establishment of High Fluoride and Tibial Fracture Model in Rats

All procedures involving rats and experimental protocols were approved by Institutional Animal Care, and the animal study was reviewed and approved by the Ethical Committee of The First Affiliated Hospital of Chongqing Medical University. Thirty male Sprague–Dawley rats aged 6 months and weighing 273 ± 16 g (Experimental Animal Center) were allocated to two experimental groups and one control group. According to the standard skeletal fluorosis rat modeling method, dental fluorosis was used as the verification standard. After the skeletal fluorosis rat model was completed, the tibia of the rat was fractured. After the X-ray was completed, the femur of the rat was measured for bone fluoride. Sodium fluoride (NaF) was included in the drinking water of each experimental group for 4 weeks at different concentrations (5.0 and 10 mM NaF). All groups were maintained in plastic cages in a room with a 12-h light/dark cycle and an ambient temperature of 21°C and were allowed ad libitum access to water. After 4 weeks of treatment, fluorosis in rat incisors was detected (Houari et al., 2014). All of the rats were anesthetized, and the hair was removed from the left hind limb. An incision was made in the skin over the medial aspect of the proximal tibia. Soft tissue was cleared from the distal end of the tibial crest, and three-point bending pliers made a blunt fracture at the upper third of the tibia. Then, a needle (0.5 mm in diameter) was inserted perpendicular to the tibial plateau and along the longitudinal axis of the tibial myeloid cavity, through which the fracture is fixed. Penicillin 1.6 million units intramuscular injection for 3 days was used to prevent postoperative inflammation. The fracture was observed by X-ray at 0 and 7 days after the operation. When rats were sacrificed, the femurs of control and test animals were dissected and were reduced to ash in porcelain crucibles at 550°C in a muffle furnace overnight. Using 100-mg samples and a constant volume with 0.1 M NaOH, we measured the fluoride concentration, and it was measured by using direct potentiometry. A minimum of five rats were used per group (Hall et al., 1972; Alexander et al., 2011; Ameeramja et al., 2018; Balkanlı et al., 2020).

### Micro-CT Analysis and Histological Analysis

For micro-CT analysis, SkyScan1174 x-ray microtomography (Bruker Company, Belgian) with an isotropic voxel size of 10.0 μm was used to image the whole tibia. Scans were conducted in 4% paraformaldehyde and using an X-ray tube with a potential of 60 kV, an X-ray intensity of 166 μA, and an exposure time of 1700 ms. The scan axis coincided with the diaphyseal axis of the tibia. For mineral formation analysis of the callus during fracture healing, a minimum of 630 slices (630 × 10.0 μm) was chosen so that the entire fracture callus was included. NRecon software was used for 3D image reconstruction, and 3D and 2D analyses were performed using software CT-AN. The callus volume (CV), bone mineral density (BMD), and bone volume/callus volume (BV/CV) were measured as previously described (Schlundt et al., 2018). All images presented are representative of the respective groups.

For the bone histological analysis, the femurs were dissected and fixed with 4% paraformaldehyde for 48 h. The femurs were then decalcified by a daily change of 10% tetrasodium EDTA for 4 weeks. Tissues were dehydrated by passage through an ethanol series, cleared twice in xylene, embedded in paraffin, and sectioned into 8-μm slices along with the coronal plate from anterior to posterior. Decalcified sections were stained with H&E and safranin O-fast green staining as previously described (Li et al., 2020a; Xiao et al., 2021).

### Immunohistochemistry (IHC)

Paraffin slides were dewaxed and hydrated as previously described, and immunohistochemical analysis was performed with heat-induced antigen retrieval in sodium citrate buffer with pH = 9 (Servicebio). Primary antibodies used were anti-OPN at 1:200 (Osteopontin, Abcam), anti-Col1 at 1:200 (Collagen 1a1, Abcam), anti-VEGF at 1:200 (Abcam), anti-CD31 at 1:100 (CST), anti-CD86 at 1:100 (Servicebio), and anti-CD206 at 1:100 (Servicebio). A biotin-labeled secondary antibody was used with the immunohistochemical kit (Servicebio), and nuclei were counterstained with hematoxylin. Follow the immunohistochemical staining method, replace the primary antibody with PBS, and perform negative control staining. The immunohistochemical staining results were observed by Olympus microscopy, and the images were analyzed using ImageJ software (Zhao et al., 2017; Dou et al., 2018).

### Cells Culture and Macrophage Polarization

RAW264.7 cells, C3H10T1/2 cells, and human umbilical vein endothelial cells (HUVEC) were obtained from the American Type Culture Collection (ATCC) and cultured in Dulbecco’s modified Eagle’s medium (DMEM) supplemented with 10% fetal bovine serum (FBS), 100 U/ml penicillin (Gibco), and 100 U/ml streptomycin (Gibco) in a 37°C incubator under a 5% CO_2_-enriched atmosphere (Zhao et al., 2017; Ye et al., 2018). The M1 differentiation was induced with LPS (100 ng/ml, Sigma), and M2 differentiation was induced with IL-4 (20 ng/ml, Sigma), while both of them were treated with the corresponding concentration of NaF for 3 days. The RAW264.7 cells were induced with LPS (100 ng/ml, Sigma) to M1 type differentiation for 24 h, and M2 differentiation was induced with IL-4 (20 ng/ml, Sigma). (Kimbrough et al., 2018; Ye et al., 2018).

### CCK-8 Assays

To determine the change in cell proliferation ability with passaging, we used the Cell Counting Kit-8 (CCK-8, MedChemExpress) to test RAW cells after a fluoride treatment at 12, 24, and 48 h. Then we used CCK-8 to test C3H10T1/2 cells and HUVEC cells 24 h after fluoride treatment. The results were recorded by a microplate reader (Thermo Scientific™, United States) at an absorbance of 450 nm. The growth curves were drawn, and the cell proliferation activity was analyzed.

### RNA Isolation and Quantitative PCR (qPCR)

Total RNA was purified from cells in 60-mm dishes using TRIzol (Invitrogen) according to the manufacturer’s instructions. Then, cDNA was obtained from total RNA extracted from cells using a reverse transcription (RT) reaction kit (TaKaRa, Japan). The cDNA level was determined by SYBR Premix Ex Taq™Ⅱ (TaKara, Japan), and the procedure was carried out as follows: 95°C for 30 s for one cycle, 95°C for 5 s, and 60°C for 30 s, followed by plate reading for 40 cycles. The PCR primers (Supplementary Table S1) were designed using Primer3 plus. The relative expression levels of the mRNAs in the groups were analyzed using the 2ΔΔCT method.

### Enzyme-Linked Immunosorbent Assay

The concentration of CCL2, CXCL12, IL-8, OSM, VEGF, and TGF-β in the supernatants of fluoride-treated RAW cells was examined by the enzyme-linked immunosorbent assay (ELISA) (Neobioscience, China).

### Immunofluorescence Stain Assay

RAW cells were seeded onto sterile coverslips in a Corning 24-well culture plate at a density of 10^4^ cells/mL and treated according to the experimental design. At 24 h after induction, cells were washed three times with PBS for 5 min each, then fixed with 4% paraformaldehyde at 3°C for 15 min in a thermostatic water bath, washed with PBS for 5 min each, and then permeabilized using 0.4% Triton X-100 for 30 min at 37°C. After cells were blocked with goat serum for 30 min, cells were incubated with the primary anti-CD68 (Zen-Bioscience), anti-CD86 (Abcam), and anti-CD163 (Abcam) overnight, followed by incubation with the corresponding fluorophore-conjugated antibodies (Abcam) for 60 min. Then cells were washed with PBS for 5 min and stained with DAPI for 5 min. The coverslips were carefully removed and then mounted on slides with glycerol. The same protocol was performed in the negative control groups except that the primary antibodies were omitted. The immunofluorescence staining results were observed by inverted fluorescence microscopy (Olympus) and quantified using ImageJ software (Wasnik et al., 2018; Gaojian et al., 2020).

### Co-Culture Assay

A co-culture assay was performed in a 24-well Corning Transwell chamber using a porous polycarbonate membrane with a pore size of 8 μm (Thermo Fisher Scientific) (Zhao et al., 2017). Briefly, in the migration analysis of mesenchymal stem cells (MSCs), RAW cells were treated with fluoride for 24 h and then treated with serum-free DMEM for another 24 h. Subsequently, this cell supernatant was seeded in the lower chambers, and a number of 10^4^ MSCs in a complete medium were seeded in the upper chambers. After 24 h of co-culture, the membranes were fixed with 4% formaldehyde and stained with 0.5% of crystal violet (Sigma). The numbers of migrated cells in the membrane in more than three randomly chosen microscopic fields were counted and averaged. In the wound healing assay, after scratching the cells, the cells were cultured for 24 h in the fluoride-treated RAW supernatant (Medina et al., 2006; Jiang et al., 2020b). In addition, this supernatant was also used in the tube formation analysis and MSC osteogenic differentiation assays.

### Tube Formation Analysis

Matrigel™ basement membrane matrix (BD Biosciences, CA, United States) was thawed at 4°C, pipetted into pre-cooled 48-well plates, and incubated at 37°C for 30 min. After Matrigel polymerization, HUVECs (6 × 10^4^ cells) were suspended in a medium and were seeded onto the Matrigel, and each condition in each experiment was assessed in triplicate. Images were taken after 6 h using a ×4 objective. The tube formation assay was analyzed and interpreted using ImageJ software based on different parameters such as mesh number, total branching length, total segment length, and so on. The total segment length was chosen as the statistical parameter.

### Osteogenic Differentiation Assays

C3H10T1/2 cells were cultured in the mouse’s bone marrow MSCs osteogenic differentiation medium (Cyagen) for 7 days. After osteogenic differentiation, the cells were co-cultured with fluoride-treated RAW cells.

### Alkaline Phosphatase Staining and Activity

For alkaline phosphatase (ALP) staining, cells were fixed with 4% paraformaldehyde for 30 min. The cells were then washed twice with PBS and stained using the BCIP/NBT Alkaline Phosphatase Color Development Kit (Beyotime). Staining was observed under a bright-field microscope after 30 min.

For the measurement of ALP activity, cells were washed twice with PBS and lysed with 150 µL of NP‐40 lysis buffer (Beyotime). The cell lysates were quantified by an alkaline phosphatase assay kit (Beyotime) using p‐nitrophenyl phosphate (pNPP) as the substrate. In the presence of magnesium ions, pNPP was hydrolyzed by phosphatases into phosphate and p‐nitrophenol. The rate of p‐nitrophenol liberation was proportional to ALP activity and was measured photometrically. The ALP activity was measured by a microplate reader (Thermo Scientific™, United States) at an absorbance of 405 nm.

### Alizarin Red S Staining

Subconfluent cells were treated with the bone morphogenetic protein 2 (BMP2) for 2 days. The cells were cultured in the presence of ascorbic acid (50 mg/ml) and b-glycerophosphate (10 mM) for 14 days. The mineralization nodules were assessed by alizarin red S staining. Briefly, the cells were fixed with paraformaldehyde at room temperature for 10 min and washed with PBS (pH adjusted to 4.2). The fixed cells were incubated in a 37°C incubator with 2% alizarin red S for 10 min, followed by careful washing with distilled water. The calcium deposits were observed under a microscope. For quantification, alizarin red S was dissolved in 10% acetic acid and the absorbance was detected at 405 nm with a microplate reader.

### Statistical Analysis

All the data are representative of at least three experiments performed in triplicate that yielded similar results unless otherwise indicated. All the quantitative experiments were performed in triplicate and/or repeated through three independent batches of experiments. The statistical analyses were performed using the software package SPSS 14.0 and by one-way analysis of variance and Student’s t-test to determine the significance of differences between results, with *p < 0.05 and **p < 0.01 regarded as significant (Zhao et al., 2017).

## Results

### 1 Construction and Evaluation of the Rat High-Fluoride Diet Model

Three groups of 10 rats each were administered 0 (control), 5.0, and 10 mM NaF (0–200 ppm) orally (in drinking water) for 4 weeks. This period was chosen to ensure the impregnation of the bone and mandibular incisor with NaF. Control mandibular incisor enamel was homogeneously colored yellow to orange, and the 5 mM NaF (100 ppm) group treated rats had discolored enamel incisors. At 10 mM NaF (200 ppm), the orange coloring completely disappeared, and the entire mandibular incisor was white and opaque. (Supplementary Figure S1A). According to the degree of dental fluorosis, as previously described, the enamel scored 0 points for normal, 1 for mild, and 2 for severe fluorosis (Ameeramja et al., 2018) (Supplementary Table S2). In the 5 mM NaF group, 40% of the rats were scored as 1, and 20% were scored as 2. In the 10 mM NaF group, 30% of the rats were scored as 1 and 50% were scored as 2 (Supplementary Figure S1B).

The fluoride concentration in the bone was estimated following the Medina et al. method (Wasnik et al., 2018), and the result showed it was significantly higher in the 10 mM and the 5 mM groups than in the control group (Supplementary Figure S1C).

### 2 NaF Inhibits Multiple Phases of Endochondral Bone Healing *in vivo*

In this study, we first investigated the effects of NaF treatment on fracture repair. Accordingly, each group of rats was subjected to fracture surgery (Supplementary Figure S2) and was observed by X-ray at 0 and 7 days after the operation (Supplementary Figure S3).

**FIGURE 1:**
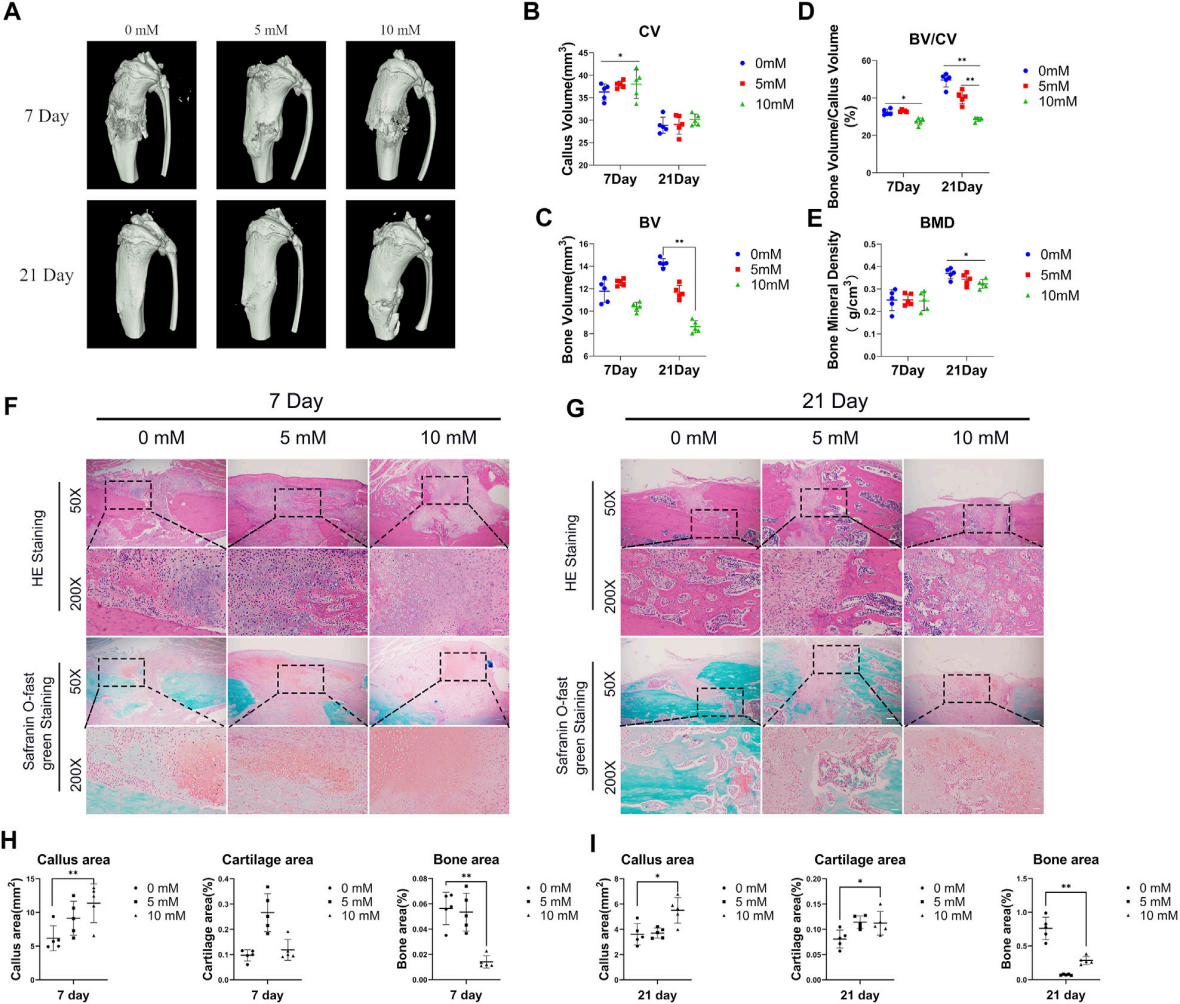
Excessive fluoride intake impaired fracture healing. **(A)** The tibia was subjected to micro-CT analysis. Representative micro-CT 3D images are shown. **(B,C,D,E)** Quantification results of the relative values of BV, CV, BV/CV, and BMD were analyzed. These data are shown as mean ± SD repeated in triplicate, *p < 0.05; **p < 0.01. **(F,G)** H&E staining and safranin O-fast green staining were performed to determine the callus area, cartilage area, and bone area (magnification ×50, scale bar = 400 μm; × 200, scale bar = 100 μm). Representative images are shown. **(H,I)** The areas of callus, cartilage, and bone were quantified using ImageJ. *p < 0.05, **p < 0.01, *t*-test, *n* = 3.

Moreover, we performed the micro-CT analysis on days 7 and 21 after fracture surgery (Figure 1A). Our data show that compared with the control group and the 5 mM group, the CV in the 10 mM group was relatively increased on day 7 (Figure1B). Meanwhile, the BV was significantly decreased in the fluoride-treated group (Figure 1C). The 1 mM group displayed approximately 5% reduction in the ratio of BV/CV on day 7 and approximately 20% reduction on day 21 compared with the control group (Figure 1D). The BMD of the fracture site in the 10 mM group on day 21 was also decreased compared with the other group (Figure 1E).

Moreover, the H&E staining and the safranin O-fast green staining were performed to detect the histology of fractured bones. The results showed that the fractures of the 10 mM group, when compared with the control group, displayed bigger fracture gaps and fewer new bones at fracture sites both at 7 and 21 days (Figures 1F, G). Accordingly, we used ImageJ software to calculate the callus area and the new bone area of the fracture gaps, and the results showed that the callus area was increased in the 10 mM group and the new bone area was decreased compared with the 0 mM group (Figures 1H, I). It was consistent with the results of micro-CT. Meanwhile, we calculated the proportion of cartilage area at the fracture gap, and the results showed that the proportion increased at 21 days in the 10 mM groups compared with the 0 mM group.

**FIGURE 2:**
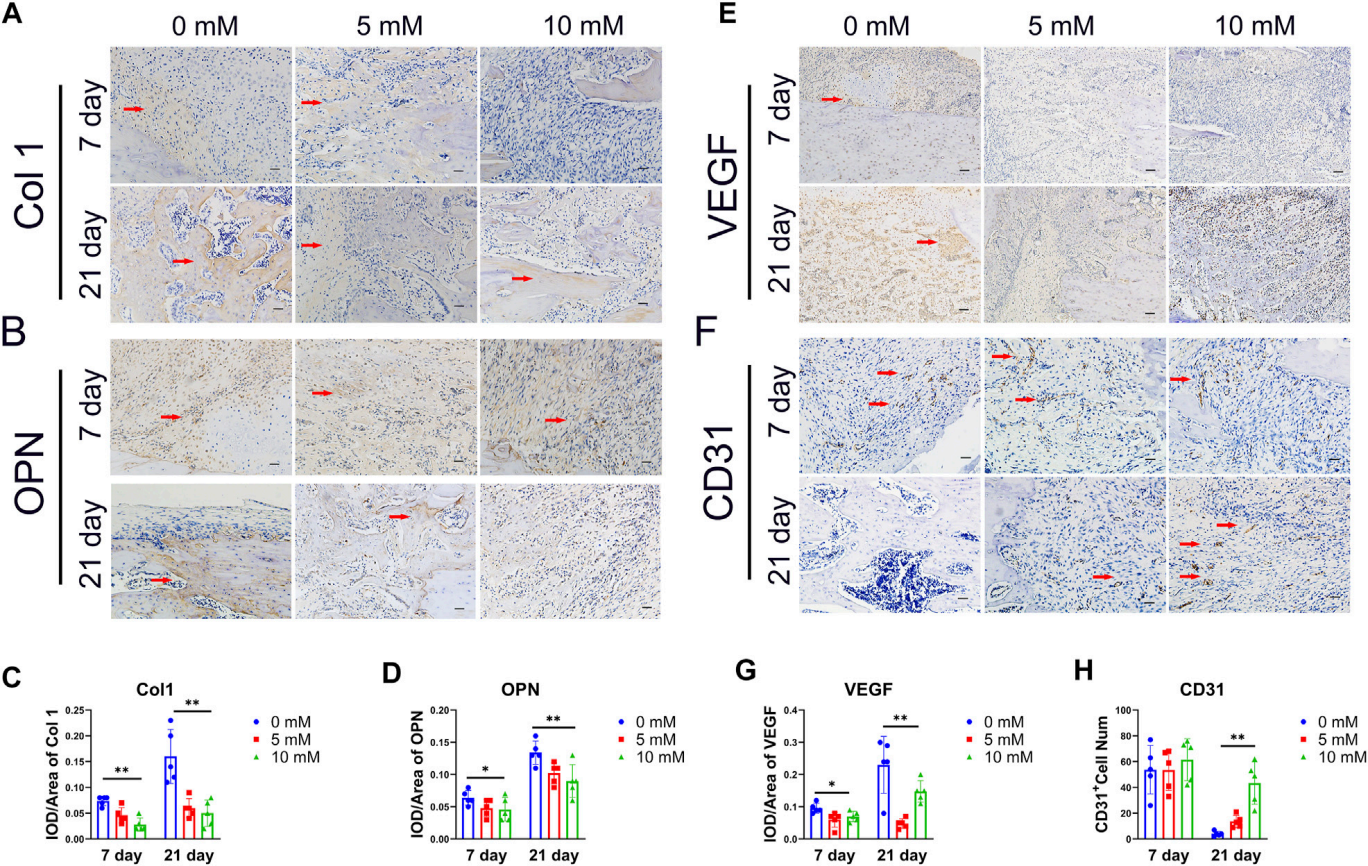
Histological analysis of the callus. **(A)** Representative immunohistochemical (IHC) stain images of the osteogenic relative marker of Col1a1 and OPN on day 7 and day 28.(× 200, scale bar 200 μm). **(B)** IHC scores of Col1a1 and OPN were quantified. **(C)** Angiogenesis markers VEGF and CD31 were detected by IHC.(× 200, scale bar 200 μm) and **(D)** quantified by ImageJ. The CD31-positive cells are indicated by arrows, and Col 1, OPN and VEGF-positive regions are indicated by arrows. The data in the figures represent the averages ± SD. Significant differences are indicated as *p < 0.05 or **p < 0.01, *t*-test, *n* = 3.

It is believed that the condition of the local vasculature and the osteoblast-associated protein are critical determinants of the outcome of fracture healing. Both are the main features of the regenerative stage. The osteogenesis markers of Col1 and OPN and the angiogenesis markers of CD31 and VEGF were detected by immunohistochemical staining. The results showed that the Col1 and OPN positive IOD/area were significantly reduced in the fluoride-treated group, especially at 21 days (Figures 2A,B). The VEGF-positive IOD/area was also reduced in the 10 mM group compared with the control group (Figure 2C). However, the CD31-positive cells were decreased in the control group compared with the 10 mM group at 21 days, which indicates that the high level of fluoride in the bone prolonged the proinflammatory stage (Figure 2D). At the same time, negative control of immunohistochemistry was used as a comparison (Supplementary Figure S4).

### 3 Rats on a High-Fluoride Diet Have a Delayed Proinflammatory Stage of Fracture Healing

**FIGURE 3:**
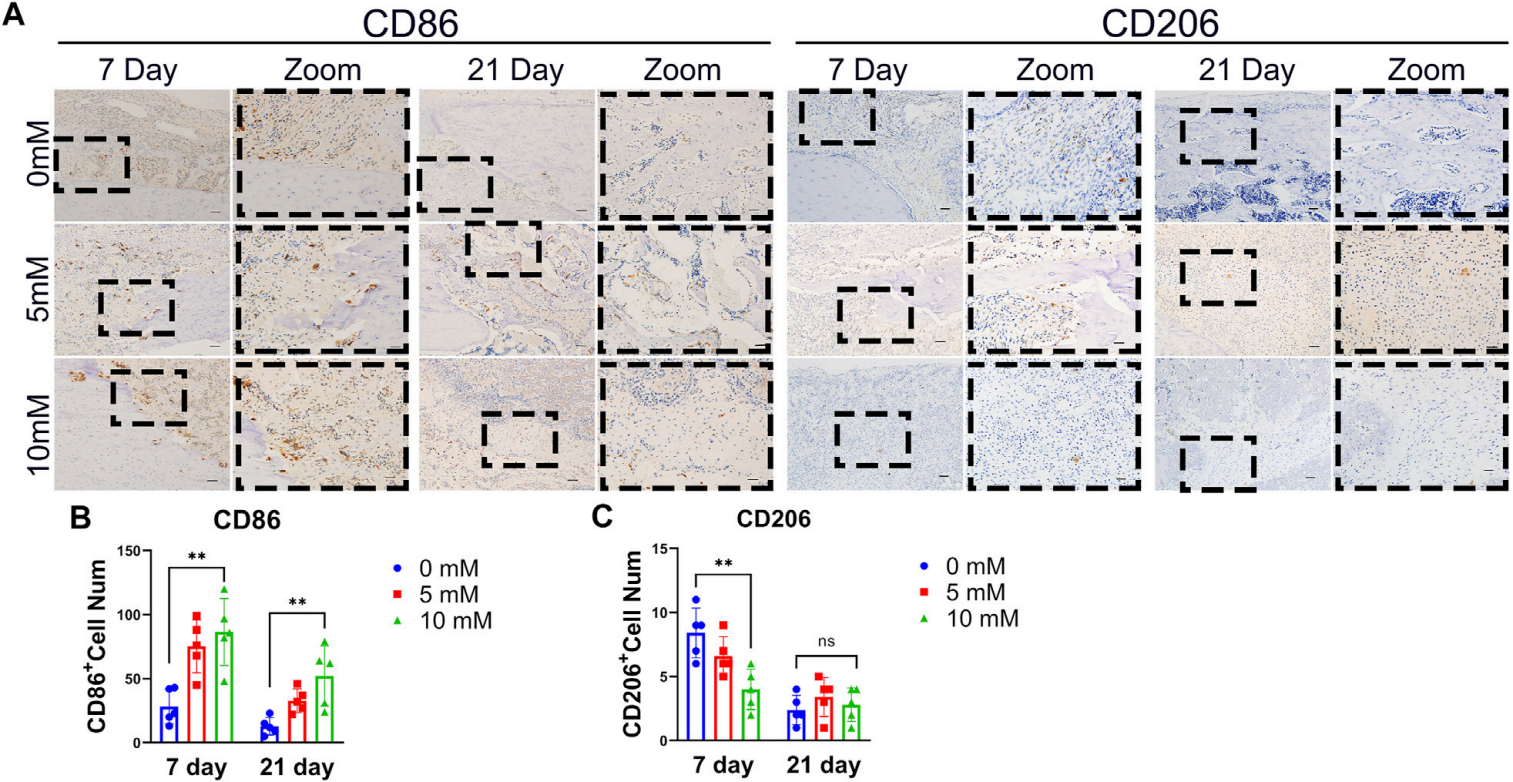
Excessive fluoride intake rats showed suppressed M2 macrophage differentiation at fracture sites. **(A)** M1 macrophage marker of CD86 and CD206 was detected by IHC at 7 and 21 days after fracture. The bar represents 200 μm (left) and 50 μm (right). **(B)** Relative positive cell numbers were detected. The data in the figures represent the averages ± SD. Significant differences are indicated as *p < 0.05 or **p < 0.01, *t*-test, *n* = 3.

It has been reported that M1 macrophages present during the early stages of fracture repair contribute to bone formation and that M2 macrophages can contribute to bone formation during the later stages of fracture healing (Schlundt et al., 2018). However, there are no reports about the influence of fluoride on M1 and M2 differentiation of macrophages at the fracture site. We detected the M1 macrophage markers CD86 and the M2 macrophage markers CD206 by immunohistochemical staining. The results showed that the number of CD86-positive cells increased with the increase in fluoride intake, and the number of CD206-positive cells decreased significantly at 7 days (Figures 3A,C). At 21 days after fracture surgery, compared with the fluoride-induced group, the number of macrophages at the fracture site was significantly reduced in the control group. Meanwhile, the existing macrophages in the fluoride-treated group were predominantly M1 (Figure 3B). Our data indicate that fluoride inhibits the M2 differentiation of macrophages early at fracture sites, leading to the prolongation of the proinflammatory stage.

### 4 Proliferation and Polarization Gene of RAWs With Different Concentrations of Fluoride

**FIGURE 4:**
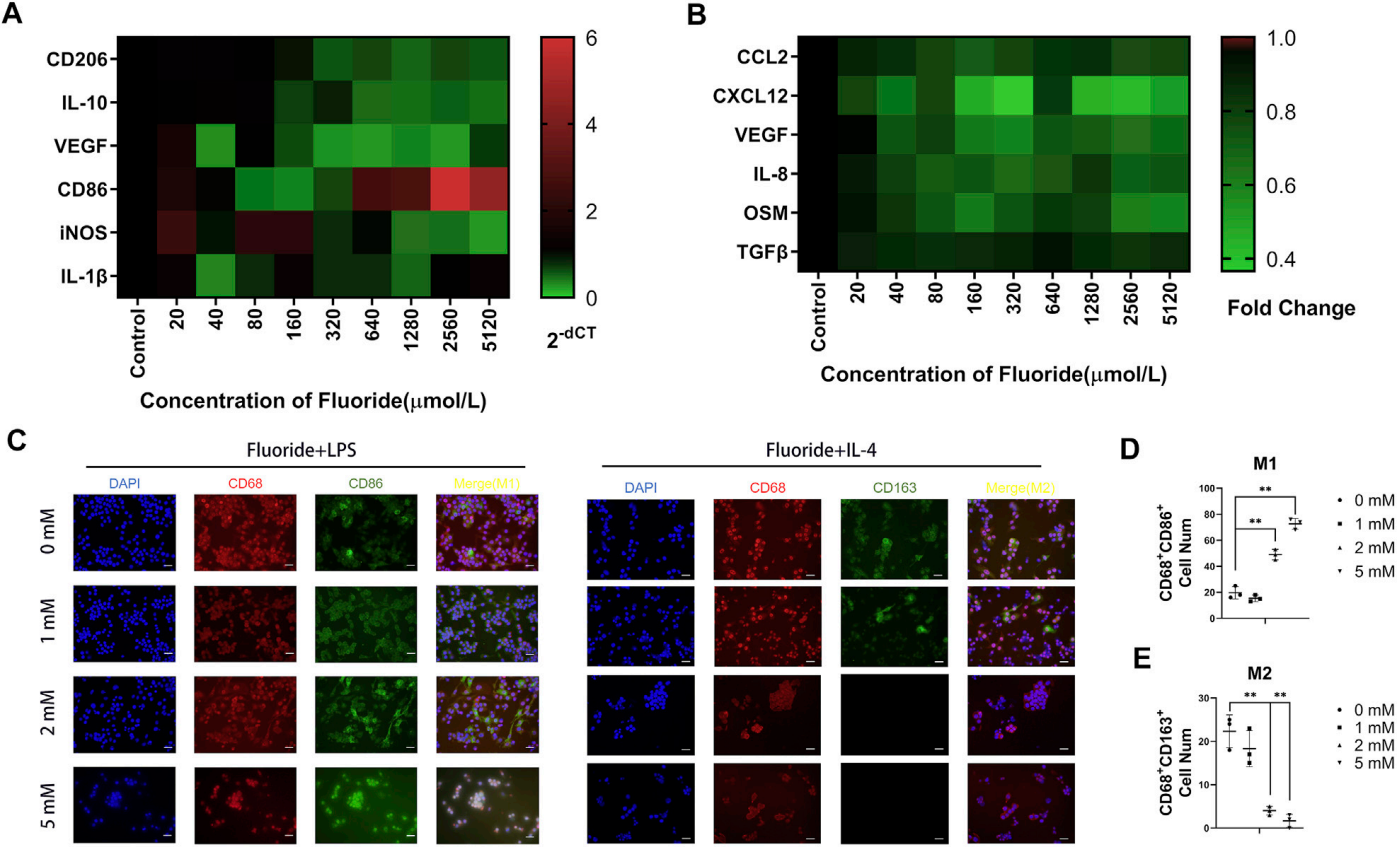
Fluoride suppressed macrophage polarization *in vitro.* **(A)** RT-qPCR assay was performed to determine the expression of the M1-relative gene, including CD86, IL-1β, and iNOS. Meanwhile, the M2-relative gene CD206, IL-10, and TGF-β were detected. **(B)** Quantification of CCL2, CXCL12, OSM, IL-8, VEGF, and TGF-β protein levels on the medium supernatant of fluoride-treated RAW by ELISA. Data were collected at 24 h after fluoride treatment. **(C)** Immunofluorescence staining was used to determine the fluoride-treated RAW, including CD86 (green) and CD206 (green), CD68 (red), and DAPI (blue) (magnification of up images = ×400, scale bar = 50 μm). **(D,E)** Immunofluorescence staining quantified analysis. Optical density by ImageJ. *p < 0.05, **p < 0.01, *t*-test. Each experiment was carried out in triplicate.

The RAW cells grew well in the complete medium with 0, 20, 40, 80, 160, 320, 640, 1,280, 2,560, 5,120, and 10,240 μM fluoride at 24 and 48 h (Supplementary Figure S6). The CCK-8 assay showed significantly decreased cell vitality in the 10,240 μM group at 24 and 48 h, whereas significantly decreased cell vitality was also found at 48 h in the 5,120 μM group. We took 24 h as experimental processing time, and further, we studied the expression of macrophage polarization-related genes under the action of various fluorine ion concentrations. The M2 type macrophage gene CD206 was significantly decreased in 1280, 2560, and 5120 μM groups (Figure 4A). Meanwhile, the M1-relative gene CD86 was increased in 1,280, 2,560, and 5,120 μM groups. The inflammation-related genes showed no significant changes (Figure 4A). Therefore, a concentration ranging from 1,000 to 5,000 μM was used for subsequent experiments.

To better understand the cytokine level of fluoride-mediated macrophages, the bone fracture healing related-cytokines secreted by macrophages were examined. The expression of the migration-related genes CCL2 and CXCL12 were significantly downregulated as fluoride concentration increased (Figure 4B). The angiogenesis factors VEGF and IL-8 were found to be significantly downregulated in fluoride concentrations of more than 40 μM (Figure 4B). There was no significant change in TGF-β (Figure 4B).

### 5 Fluoride Suppressed M2 Differentiation but Augmented M1 Differentiation of Macrophages *In Vitro*

To further confirm the function of fluoride during macrophage M1- and M2-type differentiation *in vitro*, we stimulated the RAW cells with LPS or IL-4 for M1 differentiation and M2 differentiation, respectively (Li et al., 2020a). After fluoride stimulation, these cells underwent immunofluorescent staining, and successfully induced M1 macrophages were characterized by CD68 and CD86, whereas M2 macrophages were characterized by CD68 and CD163. Immunofluorescence staining and quantitative analysis showed an active differentiation of M2 macrophages in the control group but not in the 2 and 5 mM groups (Figures 4C, E). The number of CD86^+^ and CD68^+^ cells was significantly increased in the 2 and 5 mM groups (Figure 4D).

We concluded that the fluoride suppressed M2 differentiation but augmented M1 differentiation of macrophages *in vitro*.

### 6 Fluoride-Induced Macrophages Impair Cells Migration and Suppressed Tube Formation and MSCs Osteogenic Differentiation *in vitro*

**FIGURE 5:**
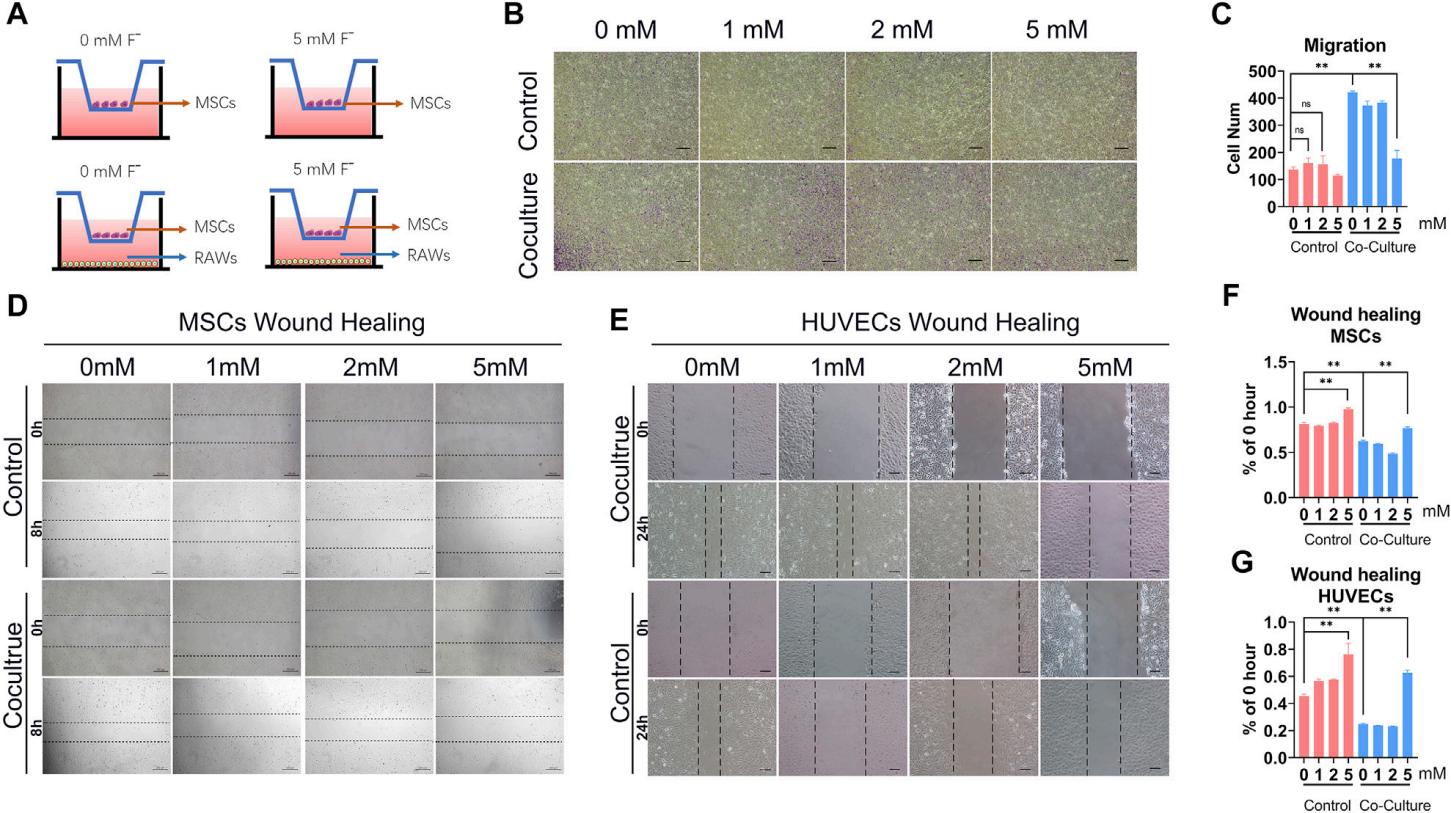
Fluoride-induced macrophages inhibit tube formation and MSCs osteogenic differentiation *in vitro*. **(A)** To investigate the effects of tube formation, the Matrigel was coated on plates and the HUVECs were adjusted to 2 × 10^5^ cells/mL/well; the supernatant medium of RAW or fluoride-treated RAW was seeded in wells. The number of tube structures was observed by light microscopy and recorded at 6 h **(B)** The tube-formation results of the quantified volume of tube area (ImageJ). **(C)** ALP activity was measured at 5 days after co-culture using ALP histochemical staining (×100, scale bar = 200 μm). **(D)** ALP quantification activity was detected at OD 405 nm. **(E)** Mineralization and calcium deposition at the late osteogenic differentiation were observed by the alizarin red S staining assay (×100, scale bar = 200 μm). **(F)** Quantification results of alizarin red S staining showed that the mineralization effect in co-culture with the fluoride-induced RAW group was poor (OD 405 nm). **(G)** RT-qPCR assay determined the osteogenic-related factors, including RUNX2 (g1) and Col1 (g2), after osteogenic differentiation. *p < 0.05, **p < 0.01, *t*-test.

Because a central bone repair mechanism in the proinflammatory stage is the recruitment of MSCs and HUVECs, we investigated the potential role of fluoride-induced macrophages in this central repair mechanism. Wound-healing and Transwell assays were performed to study the migration ability of MSCs when co-cultured with fluoride-induced macrophages (Figure 5A). In the C3H10T1/2 and HUVECs experimental groups treated with pure fluorine, the cell migration ability was significantly decreased at 5 mM. The experimental group after macrophage co-culture showed increased cell migration ability (Figures 5B–F). The wound-healing assay results in C3H10T1/2 and HUVECs also revealed that the migration was also increased in the co-cultured group (Figures 5E, G). But this enhancement of migration ability disappeared in the 5 mM group.

In order to verify the direct effect of fluoride on the viability of HUVECs and C3H10T1/2 cells, HUVECs and C3H10T1/2 cells were cultured in a complete medium containing 0, 1, 2, 5, and 10 mM fluoride for 24 h. The CCK-8 assay showed that the cell viability in 0–5 mM fluoride treatment was not significantly reduced, and the cell viability in the 10 mM fluorine treatment group was significantly reduced (Supplementary Figure S5).

**FIGURE 6:**
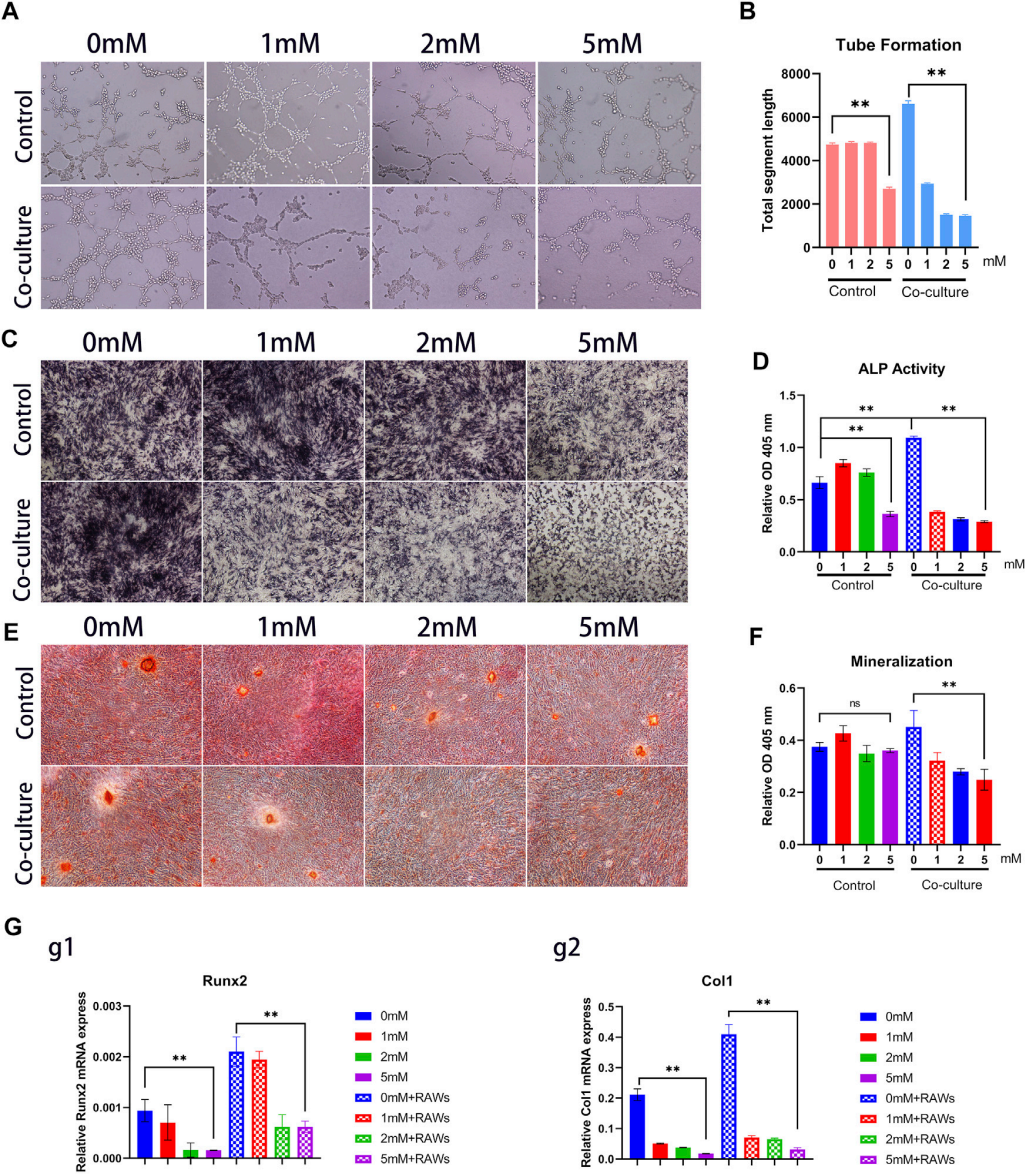
Fluoride-induced macrophages inhibit MSCs and HUVECs migration. **(A)** In a Transwell culture system, the lower chambers were added with a medium that cultured RAW or RAW + fluoride for 24 h. C3H10T1/2 cells were added to the upper chambers, and the cells were cultured. Twenty-four hours after that, the membranes were stained with crystal violet for the enumeration of migrated MSCs. **(B)** Representative microscopic images show the migration of MSCs in the membranes. The violet color indicates migrated cells (×50, scale bar 400 μm). **(C)** Cumulative data show migrated MSCs (average number of 3 different microscopic fields). **(D,E)** Wound healing assays of C3H10T1/2 and HUVECs showed significantly increased migratory abilities in co-cultured with fluoride treatment. **(F,G)** Relative rate of % of 0 h gap area. The data are shown as mean ± SD for three separate experiments. *p < 0.05, **p < 0.01.

The ability to induce angiogenesis *in vitro* in the fluoride-induced macrophages was detected by the HUVECs tube formation assay. The result revealed that although co-culture with macrophages increased tube formation, co-culture with fluoride-induced macrophages markedly decreased the tube area (Figure 6A,Supplementary Figure S7). The volume of the tube area in the 5 mM + RAW group was lower, although it showed a 0.15-fold change than that in the 0 mM + RAW group (Figure 6B).

To determine whether macrophage differentiation induced by fluoride regulates the osteogenic differentiation MSCs *in vitro*, the C3H10T1/2 cells were cultured in an osteogenic differentiation medium for a predetermined time before being co-cultured with macrophages. Then these cells were cultured in macrophage medium supernatant with or without fluoride. We found that ALP activities were significantly inhibited by the fluoride-induced macrophage medium group compared with other groups on day 3, both qualitatively and quantitatively (Figures 6C, D) Similarly, matrix mineralization was significantly inhibited on day 14 when cells were co-cultured with fluoride-induced macrophages (Figure 6E, F). When the expression of osteogenic markers was assessed, we found that Runx2 was dramatically reduced by co-culture with fluoride-induced macrophages (Figure 6G, Figure g1). Accordingly, the expression of late osteogenic markers such as Col1 was significantly decreased on day 7 (Figure 6, Figure g2). Taken together, these results indicate that fluoride-induced macrophages not only impair HUVEC tube formation but also significantly diminish BMP2-induced MSC osteogenic differentiation.

## Discussion

SF is still one of the most serious public health problems in the world and is prevalent in 50 countries such as some areas of South Asia, India, and Bengal (Johnston and Strobel, 2020; Zhang et al., 2020). However, the current treatment of SF is still not satisfactory because of the complex pathological process and secondary bone trauma such as fractures (Phipps et al., 2000; Srivastava and Flora, 2020). The prevalence of bone fractures associated with high fluoride ingestion has been debated in the past. Previous clinical evidence suggested that fluoride may improve BMD (Ringe and Rovati, 2001), although current studies suggest that this denser bone may be more brittle and susceptible to fracture (Haguenauer et al., 2000). Another report suggested that fluoride exposure in sheep may aggravate bone loss and cause fragility fractures (Hillier et al., 2000; Li et al., 2001; Simon et al., 2014). These studies tend to support the notion that high fluoride ingestion increases the risk of bone fracture. Meanwhile, fluoride could be incorporated into apatite crystals and lead to the formation of fluorapatite (Simon et al., 2014). Fluorapatite may result in higher resistance to bone resorption by osteoclasts, and its accretion is perpendicular to the collagen fibers, unlike hydroxyapatite. This results in fewer proteins binding and more brittle bones. However, little data are available on whether this change affects the bone fracture healing process. Therefore, it is of great significance to understand the effect of high fluoride exposure on fracture healing. In this study, for the first time, we discovered that excessive fluoride exposure delayed bone fracture healing in rats. Excessive fluorine exposure mainly impairs the regenerative stage of fracture healing, as micro-CT results revealed that osteogenesis was inhibited on day 21. A growing number of fracture healing studies have shown that there is no lack of progenitor cells at the fracture site, but fracture healing is impaired, mainly due to changes in the regenerative stage of healing (Gaojian et al., 2020). Fluoride can cause inflammation in multiple systems of the body, such as the gastrointestinal tract and the heart muscle, and in bone fracture healing, dysregulated inflammation leads to suppression of bone formation (Quadri et al., 2018; Li et al., 2020b). The main feature is the change in macrophage polarization. Intriguingly, we found that the M2 macrophages were decreased in the callus of the 10 mM fluoride-treated group, which might be the cause of fracture nonunion.

Recently, the relation between macrophage polarization and bone fracture healing or bone regeneration is getting increasing attention in various pathological conditions (Schlundt et al., 2018). Particularly, in the endochondral ossification pathway of fracture healing, recent research has determined that process is based on carefully coordinated cross-talk between macrophage polarization and bone-forming cells (Houari et al., 2014; Gaojian et al., 2020; Xiao et al., 2021). Both the M1 and M2 macrophages affect multiple stages of fracture healing but in different ways. One study revealed that M1 macrophages are important for the recruitment and osteogenic priming of MSC during the proinflammatory stage (Ye et al., 2018). In contrast, some studies suggest that M2 macrophages mainly contribute to bone formation during the later stages of fracture healing (Hu et al., 2018; Schlundt et al., 2018). Macrophage differentiation of M1 and M2 were the main variables in our animal studies, with no previously published data reported on whether fluoride participated in the differentiation of macrophages into M1 and M2. In this study, we stimulated RAW with LPS or IL-4 for M1 differentiation and M2 differentiation and exposed them to fluoride stimulation, respectively. More M1 macrophages were detected by immunofluorescence assay than M2 macrophages in the high-dose fluoride-induced group. This suggests that the delay in fracture healing may be due to the inhibition of M2 differentiation by fluoride. Meanwhile, a recent study has shown that a new type of β-tricalcium phosphate ceramic promotes M1 differentiation both *in vivo* and *in vitro* but has no significant osteoinduction ability *in vivo*. This suggests that during the endochondral osteogenesis process, the persistence of M1-type macrophages in the late stage of fracture healing may lead to long-term local inflammation that affects bone regeneration (Chen et al., 2020). We found that although macrophages can promote MSCs and HUVECs migration *in vitro*, this enhancement disappears in high fluorine concentration. Meanwhile, the osteogenic differentiation and tube formation were inhibited. This further suggests that excessive fluoride-induced macrophages impair the regenerative stage of fracture healing by inhibiting MSC osteogenesis and angiogenesis of HUVECs.

The toxicity effect of fluoride in a dose-dependent manner has been previously reported (Ribeiro et al., 2017; Jiang et al., 2020a). There is still no accurate standard for fluoride ion concentration in the bone tissue of patients with SF, which has led to a blurred relationship between bone damage and bone fluoride concentration. It would be interesting to further understand the effect of fluoride concentration on bone metabolism. An *in vitro* study showed that more than 50 μM fluoride can decrease the estimated elastic modulus in the 3-point bending assay (Rezaee et al., 2020). At a range of 50–1500 μM, NaF-incubated bones had significantly greater indentation distances, higher displacement-to-maximum force, and lower estimated elastic modulus, ultimate stress, and bending rigidity with increasing NaF concentration compared to vehicle-incubated bones. However, this range of fluorine concentration is quite different from *in vivo* conditions (Rezaee et al., 2020). Our results showed that when the fluoride ion concentration in the femur is greater than 500 mg/kg, fracture healing is delayed in SD rats. *In vitro* cell studies have reported that fluoride above 16 ppm (approximately 840 μM fluoride) for 7 days can decrease the cell viability of BMSCs (Wu et al., 2019). To determine the concentration of fluorine toxicity in macrophages, we used macrophages and cultured them in the range of 10–10,240 μM fluoride. Our results showed that when the fluoride concentration was over 5120 μM, proliferation was decreased at 48 h. To clarify the relationship between fluoride concentration and macrophage polarization, we measured the polarization-relative genes in the range of 0–5120 μM fluoride. The results showed that M2-related gene expression continued to decrease with the increase in fluoride concentration. This feature was further confirmed by ELISA assays, for the downregulation of M2 macrophages, secreted cytokines VEGF and IL-8, and the expression of MSC and HUVEC migration relative cytokines (CXCL12 and CCL2 that are mainly secreted by M1 macrophages) were also decreased. These studies on fluorine concentration are helpful to further understand the pathological processes and diagnosis of fluorosis.

## Conclusion

Our results revealed that the high fluoride intake impaired bone fracture healing. The high fluoride concentration affects the polarization of macrophages, resulting in a decrease in M2 differentiation. Although this study does not directly address the cause-and-effect relationship between fluoride-induced macrophage polarization and the impairment of fracture repair, it still provides an important reference for the clinical treatment of bone fracture patients with a history of high fluoride intake or SF. Meanwhile, it also provides a reference for the pathological study and treatment of SF.

## Data Availability

The raw data supporting the conclusions of this article will be made available by the authors, without undue reservation.
