# Supplementary figures and images for: High Fluoride Ingestion Impairs Bone Fracture Healing by Attenuating M2 Macrophage Differentiation
Source: Front Bioeng Biotechnol. 2022 May 20;10:791433. doi: 10.3389/fbioe.2022.791433 (PMC9164140; doi:10.3389/fbioe.2022.791433)

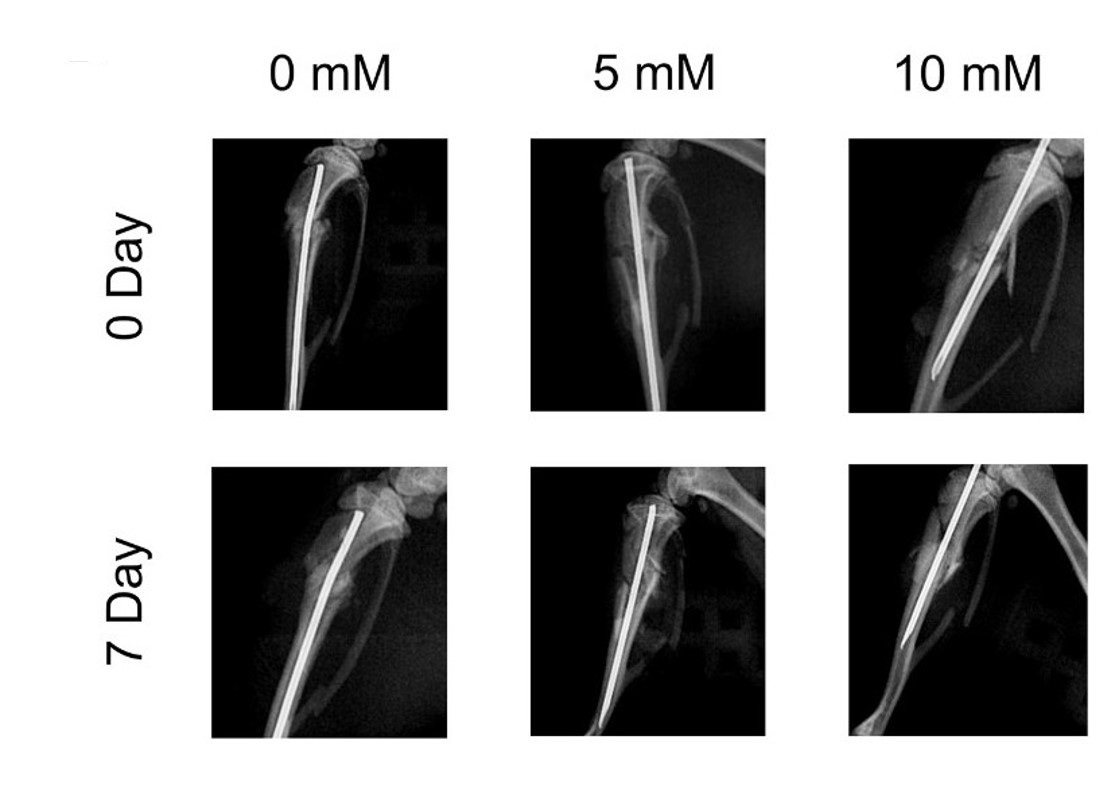

Supplement: Supplementary file 1 [file Image3.JPEG]

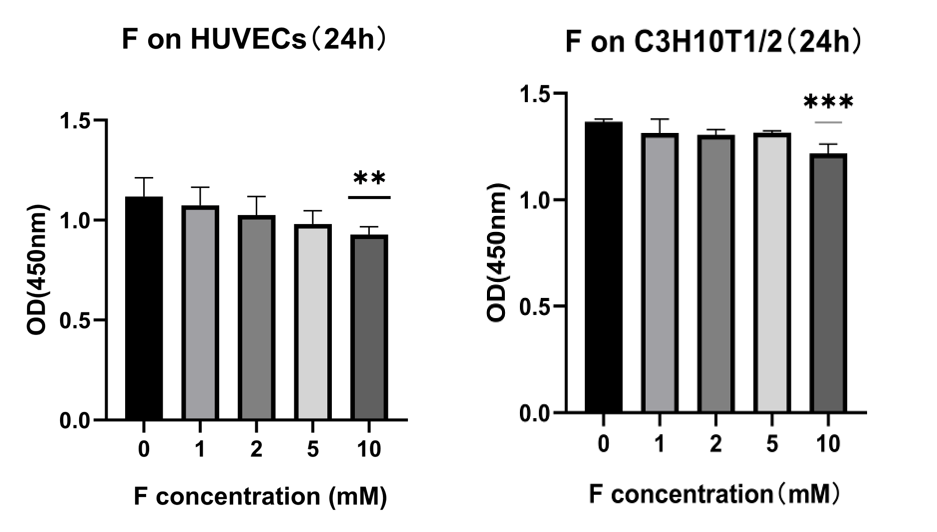

Supplement: Supplementary file 2 [file Image6.TIF]

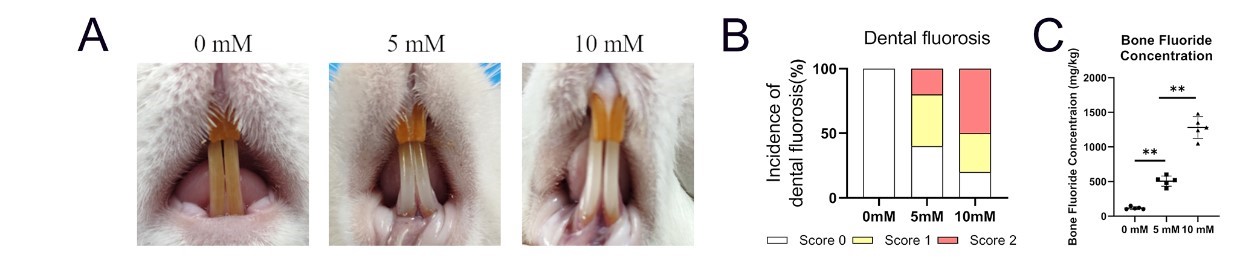

Supplement: Supplementary file 4 [file Image1.JPEG]

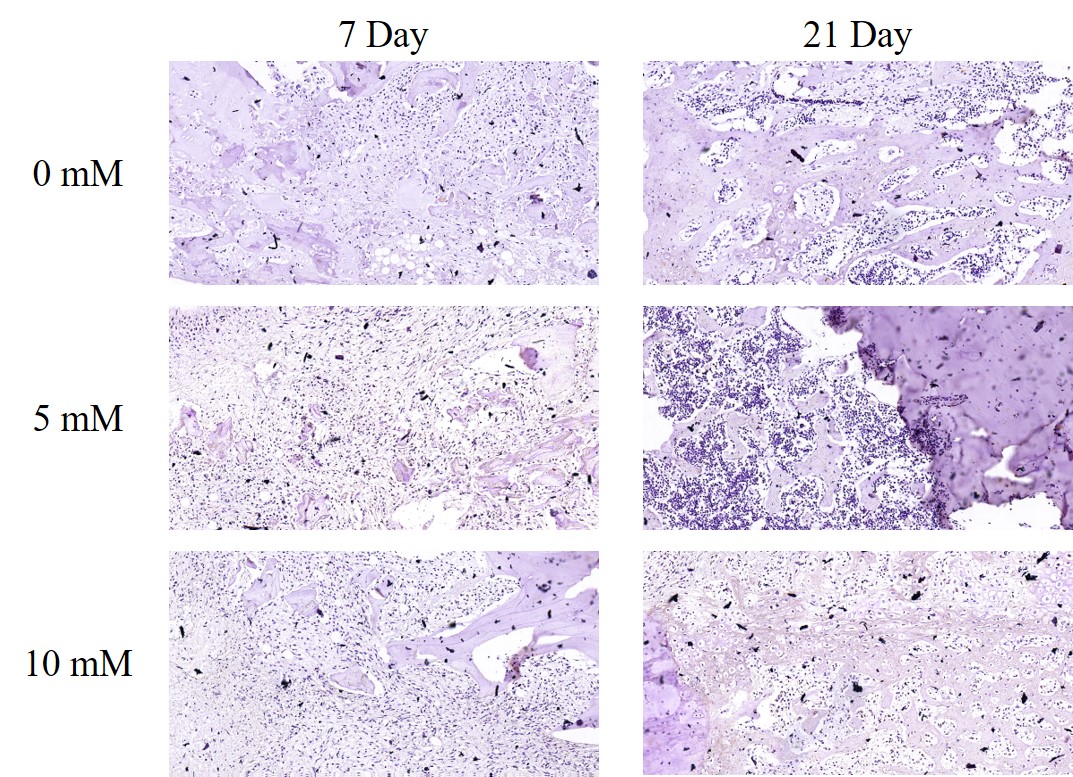

Supplement: Supplementary file 5 [file Image4.JPEG]

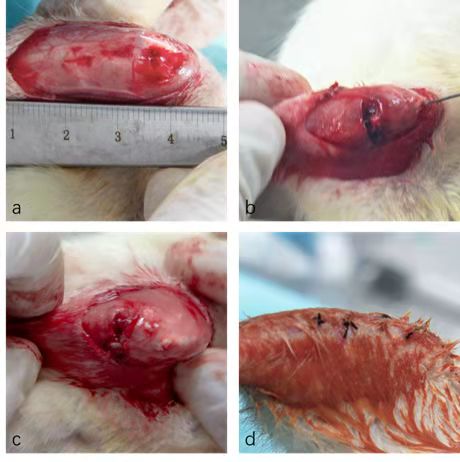

Supplement: Supplementary file 6 [file Image2.JPEG]

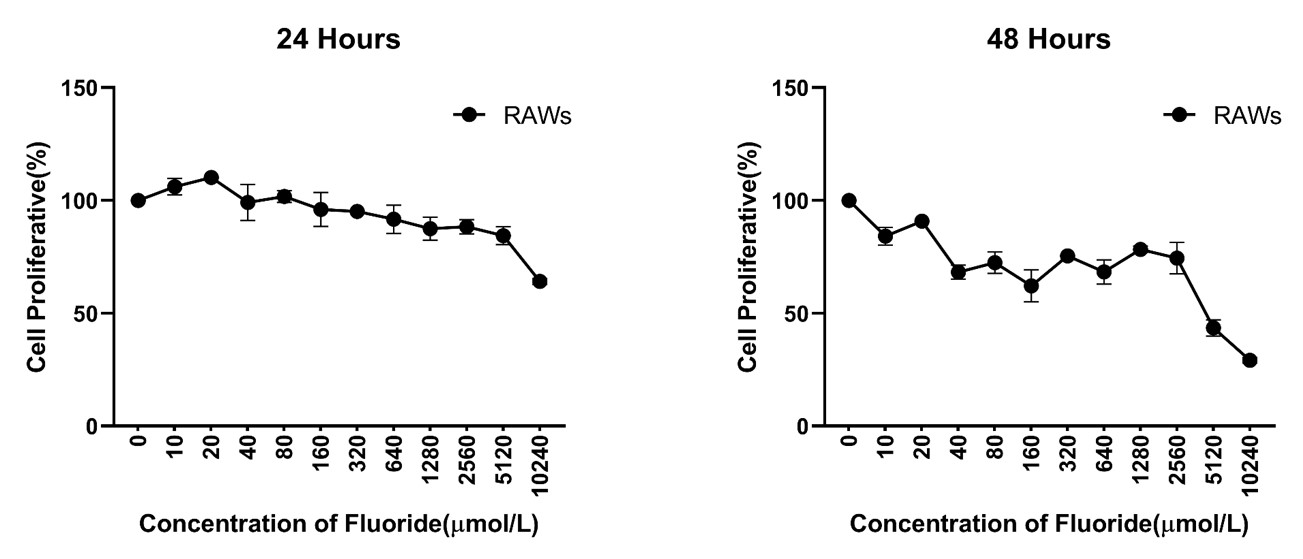

Supplement: Supplementary file 7 [file Image5.JPEG]

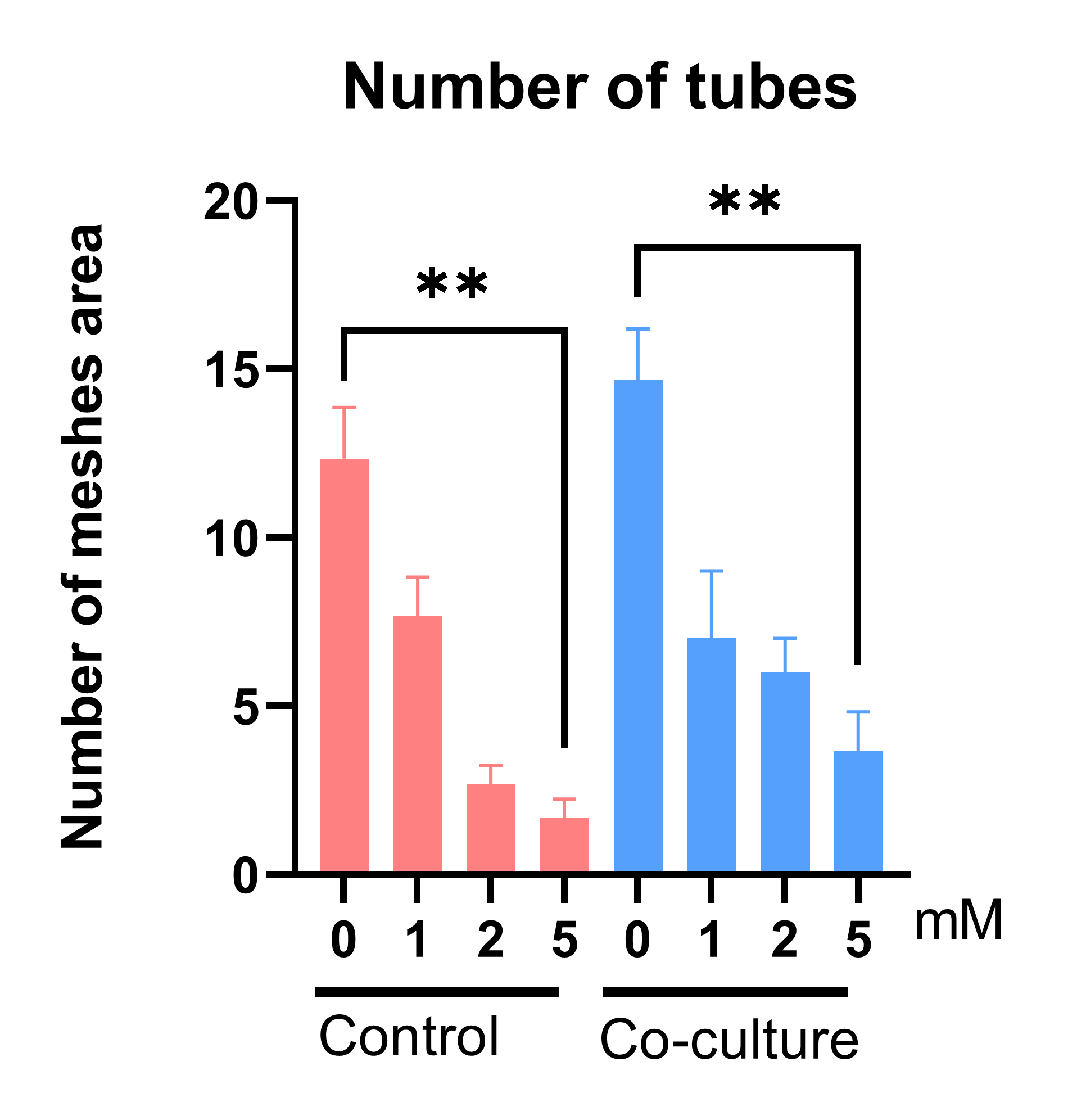

Supplement: Supplementary file 8 [file Image7.TIF]
