# Supplementary material for: High Fluoride Ingestion Impairs Bone Fracture Healing by Attenuating M2 Macrophage Differentiation
Source: Front Bioeng Biotechnol. 2022 May 20;10:791433. doi: 10.3389/fbioe.2022.791433 (PMC9164140; doi:10.3389/fbioe.2022.791433)
Supplement: Supplementary file 3 [file Table1.DOCX]

Supplementary. Table 1 Primer sequence of the target genes.

| CD86-Forward | 5′‐ACGTATTGGAAGGAGATTACAGCT‐3′ |
| --- | --- |
| CD86-Reverse | 5′‐TCTGTCAGCGTTACTATCCCGC‐3′ |
| IL-1β-Forward | 5′‐TGGACCTTCCAGGATGAGGACA‐3′ |
| IL-1β-Reverse | 5′‐GTTCATCTCGGAGCCTGTAGTG‐3′ |
| TNFα-Forward | 5′‐GGTGCCTATGTCTCAGCCTCTT‐3′ |
| TNFα-Reverse | 5′‐GCCATAGAACTGATGAGAGGGAG‐3′ |
| GAPDH-Forward | 5′‐CTATGAGGACCAGGTTGTCT‐3′ |
| GAPDH-Reverse | 5′‐TTGTCATACCAGGAAATGAGC‐3′ |
| RUNX2-Forward | 5′‐CCGGTCTCCTTCCAGGAT‐3′ |
| RUNX2-Reverse | 5′‐GGGAACTGCTGTGGCTTC‐3′ |
| CD206-Forward | 5′‐AGCCAACACCAGCTCCTCAAGA‐3′ |
| CD206-Reverse | 5′‐CAAAACGCTCGCGCATTGTCCA‐3′ |
| IL-10-Forward | 5′‐CGGGAAGACAATAACTGCACCC‐3′ |
| IL-10-Reverse | 5′‐CGGTTAGCAGTATGTTGTCCAGC‐3′ |
| TGFβ-Forward | 5′‐TGATACGCCTGAGTGGCTGTCT‐3′ |
| TGFβ-Reverse | 5′‐CACAAGAGCAGTGAGCGCTGAA‐3′ |
| Col1-Forward | 5′‐CCTCAGGGTATTGCTGGACAAC‐3′ |
| Col1-Reverse | 5′‐CAGAAGGACCTTGTTTGCCAGG‐3′ |
| VEGFA-Forward | 5′‐CTGCTGTAACGATGAAGCCCTG‐3′ |
| VEGFA-Reverse | 5′‐GCTGTAGGAAGCTCATCTCTCC‐3′ |
| iNOS-Forward | 5′‐GAGACAGGGAAGTCTGAAGCAC‐3′ |
| iNOS-Reverse | 5′‐CCAGCAGTAGTTGCTCCTCTTC‐3′ |

Supplementary. Table 2 The degree of dental fluorosis.

| Score | Feature |
| --- | --- |
| 0 | incisor enamel colored yellow |
| 1 | white bands appeared |
| 2 | whole incisor appeared completely white |
